# Cell Motility Dynamics in Glaucoma: Mechanisms, Pathogenic Roles, and Therapeutic Targeting

**DOI:** 10.3390/medicina61122219

**Published:** 2025-12-16

**Authors:** Dario Rusciano, Caterina Gagliano, Alessandro Avitabile, José Fernando Maya-Vetencourt

**Affiliations:** 1 Fidia Pharmaceuticals, Ophthalmology Research, Catania University, 95100 Catania, Italy; 2 Faculty of Medicine and Surgery, University of Enna “Kore”, 94100 Enna, Italy; 3 Mediterranean Foundation “G.B. Morgagni”, 95100 Catania, Italy; 4 Neurovisual Science Technology (NEST), 95100 Catania, Italy; alessandro.avitabile2001@gmail.com; 5 Department of Biology, Physiology Institute, University of Pisa, 56100 Pisa, Italy; maya.vetencourt@unipi.it

**Keywords:** cell motility, trabecular meshwork, Schlemm’s canal, Rho/ROCK signaling, TGF-β_2_, mechanotransduction, astrocyte reactivity, endothelial–mesenchymal transition

## Abstract

Cell motility—the dynamic process encompassing migration, adhesion modulation, cytoskeletal remodeling, and extracellular matrix (ECM) interactions—is fundamental to ocular homeostasis. In glaucoma, disrupted motility of trabecular meshwork (TM) and Schlemm’s canal (SC) cells contributes to impaired aqueous humor outflow and elevated intraocular pressure (IOP), while reactive motility of optic nerve head (ONH) glial cells promotes fibrosis and neurodegeneration. Mechanistically, TM/SC motility is regulated by Rho GTPase and ROCK signaling, focal adhesion dynamics, and ECM interactions, while glial cells respond to mechanical stress and cytokines such as TGF-β_2_. Cytoskeletal alterations, ECM stiffening, and endothelial–mesenchymal transition (EndMT) contribute to glaucomatous damage by reducing normal cell motility and tissue remodeling capacity. Aberrant motility at the ONH, including heterogeneous astrocytic reactivity, leads to lamina cribrosa remodeling and retinal ganglion cell degeneration. Therapeutically, ROCK inhibitors improve TM/SC motility and outflow, suppress EndMT, and may confer neuroprotection. Stem cell-based strategies and modulation of TGF-β_2_ or mechanotransduction pathways represent emerging approaches to restore physiological motility and regenerative potential. Despite promising advances, challenges remain in ensuring targeted, durable, and safe modulation of cellular dynamics. Understanding and therapeutically harnessing cell motility offers a unifying framework to address both pressure-dependent and neurodegenerative mechanisms in glaucoma.

## 1. Introduction

Cell motility—the coordinated processes of cell migration, regulated changes in adhesion, cytoskeletal remodeling and dynamic interactions with the extracellular matrix (ECM)—is a fundamental property of many ocular cell types that underpins tissue homeostasis and adaptability. In homeostatic conditions in the eye, the trabecular meshwork (TM) and Schlemm’s canal (SC) cells continuously remodel cell–cell and cell–matrix contacts to maintain appropriate aqueous humor outflow resistance, while resident glia and supporting cells in the optic nerve head (ONH) preserve axonal architecture and respond to mechanical or metabolic changes. Perturbations in motile behaviors therefore have the potential to alter both pressure-dependent and pressure-independent pathways that may culminate in glaucomatous injury [1]. Consequently, we chose in this review to focus on cell motility because emerging evidence indicates that dysregulated cytoskeletal dynamics, adhesion remodeling, and mechanotransduction constitute a unifying mechanobiological framework that links outflow resistance with ONH vulnerability.

In the anterior segment, TM and SC cells constitute the principal cellular regulators of conventional outflow, as recently summarized in the comprehensive review by Zhou and collaborators [2]. Their ability to alter shape, form and disassemble focal adhesions, and to rearrange the actin–myosin cytoskeleton, enables dynamic modulation of inter-trabecular spaces and endothelial pores that determine facility of outflow. Pharmacological strategies that enhance cytoskeletal relaxation—such as nanoceria-assisted delivery of the ROCK inhibitor Y-27632—can potentiate these motile responses and improve outflow facility in experimental models of ocular hypertension [3]. In glaucoma, a consistent pathological signature is decreased TM cellularity accompanied by ECM accumulation and increased tissue stiffness; these changes limit the normal motile and remodeling responses of the outflow pathway and thereby raise outflow resistance and intraocular pressure (IOP) [4]. The notion that antifibrotic and pro-remodeling interventions directed at the TM/SC microenvironment can restore outflow underscores the centrality of cell motility to IOP homeostasis [1,5].

At the molecular level, paracrine mediators in the aqueous humor—above all transforming growth factor-β2 (TGF-β_2_)—drive profibrotic programs in TM and SC cells that include altered cytoskeletal architecture, enhanced ECM deposition and reduced ECM turnover. Early work by [6] first demonstrated that TGF-β_2_ directly stimulates ECM production in human TM cells, a finding later expanded by multiple studies showing elevated TGF-β_2_ concentrations in glaucomatous eyes [7]. These changes are mechanistically linked to the induction of cross-linked actin networks, enhanced cellular contractility and diminution of the motile phenotype required for normal outflow regulation. Because TGF-β_2_ engages a broad downstream network (including YAP/TAZ, focal-adhesion kinases and ECM-remodeling enzymes), it represents a convergent signal that couples biochemical and biomechanical changes to impaired cell motility [8,9].

Mechanical forces and mechanotransduction operate as powerful modulators of ocular cell behavior. TM and SC cells are subject to cyclical stretch, pressure gradients and shear forces; mechanosensitive channels and pathways translate these cues into cytoskeletal remodeling, transcriptional responses and, in some contexts, cell death. Work implicating ion channels such as PIEZO1 (and TRPV4) in TM mechanotransduction demonstrates how stretch-activated signaling can reconfigure cell–ECM contacts and influence outflow dynamics—a mechanistic bridge that links mechanical stress to altered motility and tissue degeneration. More recent studies also suggest that pathological mechanotransduction may trigger degenerative programs (for example, ferroptosis in TM cells) that reduce the pool of motile, reparative cells in the outflow pathway [10,11].

In the posterior segment, glial cells of the ONH—astrocytes, microglia and oligodendrocyte precursors—mount complex responses to IOP elevation and other stresses. Rather than a uniform, binary “reactive” state, single-cell and transcriptomic investigations reveal heterogeneity in astrocyte responses: subpopulations show process retraction or extension, differential expression of ECM and adhesion genes, and distinct phagocytic or inflammatory profiles. These heterogeneous motile and phenotypic shifts participate in lamina cribrosa remodeling and extracellular matrix deposition at the ONH, processes that can mechanically and trophically compromise retinal ganglion cell axons and accelerate neurodegeneration independent of IOP. Thus, maladaptive glial motility and ECM remodeling form an essential part of the non-pressure-only view of glaucoma pathogenesis [12,13].

Because the fundamental motile machinery is more extensively characterized in the trabecular meshwork than in the optic nerve head, TM dysfunction forms the core focus of this review. Nevertheless, glial motility at the ONH represents a mechano-biologically connected extension of the same processes, contributing to lamina cribrosa remodeling and retinal ganglion cell vulnerability.

Therapeutically, the motile machinery of TM/SC and ONH cells offers multiple entry points. Pharmacologic modulators that relax actomyosin contractility (for example, Rho/ROCK-pathway inhibitors) increase outflow facility by altering focal adhesions and cytoskeletal tension [14]; conversely, antifibrotic strategies that blunt TGF-β_2_ signaling or downstream YAP/TAZ activity may restore a more permissive ECM and enable cell migration and repair. Regenerative approaches—mobilizing endogenous TM stem/progenitor cells or delivering replacement cells—depend intrinsically on the capacity of those cells to migrate, integrate and function within a mechanically altered microenvironment; therefore, successful regeneration will likely require parallel modification of ECM stiffness and local signaling to re-establish a motility-permissive niche [1,15].

**Figure 1:**
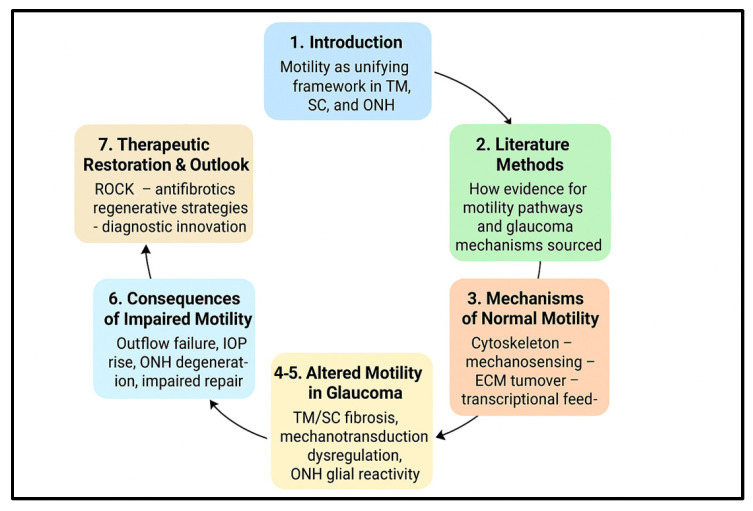
Structural overview of the manuscript within a motility-centered framework. The figure summarizes how the seven sections of the review connect along a coherent motility-centered narrative. After the Introduction (Section 1) and Methods (Section 2), the manuscript describes the Mechanisms of Normal Motility (Section 3), followed by Altered Motility in Glaucoma (Section 4 and Section 5). These changes lead to the Consequences of Impaired Motility (Section 6) and ultimately inform current and emerging approaches in Therapeutic Restoration and Outlook (Section 7). The circular layout highlights motility as the central organizing principle linking all components of glaucoma pathophysiology.

Taken together, a motility-centric perspective synthesizes molecular, mechanical and cellular levels of analysis into a coherent framework for glaucoma research and therapy. It makes explicit testable predictions: (a) that early detection of motility dysfunction or mechanotransduction abnormalities could identify eyes at risk before irreversible axonal loss; (b) that targeted modulation of motility pathways can produce durable improvements in outflow and neuroprotection; and (c) that combination strategies (mechanical/ECM modulation + cell-based repair) will be required to overcome the stiffened, fibrotic microenvironments characteristic of advanced disease. The remainder of this review unpacks these themes—examining normal motile programs in TM and SC, the maladaptive changes seen in glaucoma, the mechanotransducive drivers that link stress to dysfunction, and the evolving therapeutic toolbox aimed at restoring physiological cell dynamics (Figure 1).

## 2. Literature Search Methodology

To provide a comprehensive and current synthesis of the role of cell motility in glaucoma, a systematic literature search was conducted across major electronic databases, including PubMed, Web of Science, and Scopus, covering publications up to August 2025. The search strategy was designed to capture the multifaceted nature of cellular dynamics in glaucomatous pathology.

Core search terms encompassed key concepts central to this review:

- Cellular Motility and Cytoskeleton: “cell motility,” “cell migration,” “cytoskeletal remodeling,” “actomyosin contractility,” “focal adhesion.”
- Glaucoma Pathogenesis: “glaucoma,” “trabecular meshwork,” “Schlemm’s canal,” “optic nerve head,” “lamina cribrosa,” “retinal ganglion cell degeneration.”
- Molecular Pathways and Mechanisms: “Rho GTPase,” “ROCK signaling,” “TGF-beta2,” “mechanotransduction,” “Piezo1,” “TRPV4,” “YAP/TAZ,” “extracellular matrix stiffening,” “endothelial-mesenchymal transition.”
- Therapeutic Interventions: “ROCK inhibitor,” “netarsudil,” “ripasudil,” “stem cell therapy,” “regenerative medicine,” “senolytic,” “ferroptosis inhibitor.”

The selection process involved screening titles and abstracts to identify literature that directly addressed the mechanisms, pathogenic roles, or therapeutic targeting of cell motility in the context of glaucoma. We prioritized original research articles (including in vitro, ex vivo, and in vivo studies), clinical trials, and high-quality systematic reviews. Given the rapid advancements in ocular imaging and mechanobiology, we also included seminal studies on novel diagnostic technologies, such as optical coherence elastography and phase-sensitive OCT, where they pertained to measuring cellular-level biomechanical changes.

The final reference list for this review was curated to ensure a robust and evidence-based narrative. It includes foundational studies that established key principles, alongside cutting-edge research from 2024 to 2025 that highlights emerging therapeutic targets and diagnostic paradigms. This methodological approach ensures that the present review offers a state-of-the-art perspective on cell motility dynamics as a central axis in glaucoma.

## 3. Mechanistic and Pathophysiological Overview

### 3.1. Mechanisms of Normal Motility in the Aqueous Outflow Pathway

The conventional aqueous humor outflow pathway—the trabecular meshwork (TM) together with Schlemm’s canal (SC)—is an actively regulated, motile system whose cellular behaviors determine outflow resistance and thus intraocular pressure (IOP). The motility of TM and SC cells is driven by tightly coupled cytoskeletal dynamics, adhesion remodeling and extracellular matrix (ECM) turnover, and is continuously tuned by biochemical and mechanical cues from the microenvironment [16,17]. These dynamic interactions are underpinned by a finely regulated cytoskeletal machinery, which serves as the physical and biochemical interface translating mechanical and molecular cues into motile responses.

The subsections below are organized from the immediate, rapid regulators of motility (cytoskeletal dynamics and ion-channel mechanosensing) toward progressively slower, integrative mechanisms (ECM remodeling and mechano-transductive transcription), reflecting the biological hierarchy that governs TM and SC cell movement.

### 3.2. Cytoskeletal Regulation in TM and SC Cells

Under physiological conditions, TM cells dynamically reorganize actin filaments, myosin II–driven contractile units, and focal adhesions to alter cell shape and stiffness; these adjustments modulate the porosity of the juxtacanalicular tissue and thus outflow facility. Activation of the Rho family GTPases (notably RhoA) and their downstream effector ROCK controls stress-fiber formation, myosin-light-chain phosphorylation, and focal-adhesion assembly—molecular events that directly set cellular contractility and adhesive strength in TM cells. Classic and contemporary reviews summarize how Rho/ROCK signaling functions as a rheostat for TM stiffness and outflow regulation [16,18].

Measurements using atomic-force microscopy (AFM), a nanomechanical imaging method that quantifies tissue stiffness by scanning with a fine tip, have confirmed that TM tissue stiffness is a key biophysical determinant of outflow resistance, with glaucomatous TM exhibiting significantly increased elastic modulus values even before overt cellular loss [19]. This biomechanical context emphasizes that normal TM function relies on constant cytoskeletal adaptation to maintain optimal compliance.

SC endothelial cells, although endothelial in identity, display active cytoskeletal remodeling that underlies giant vacuole and transcellular pore formation—reversible structural specializations that permit episodic aqueous passage through the inner wall. These structures depend on coordinated actin–myosin dynamics and junctional remodeling in response to pressure and shear changes, demonstrating that SC endothelium is an intrinsically motile regulator of outflow rather than a passive conduit [17,20].

Integrin-FAK–Src signaling provides the molecular link between ECM mechanics and cytoskeletal state: engagement of integrins in a compliant ECM promotes focal-adhesion turnover and motility, while ECM stiffening enhances focal-adhesion maturation, stress-fiber formation and a less motile, contractile phenotype. Crosstalk between these adhesion pathways and mechanosensitive ion channels (see below) ensures that TM/SC cells translate mechanical stimuli into cytoskeletal and transcriptional responses [21,22]. Beyond cytoskeletal and adhesion-mediated regulation, TM and SC cells sense and respond to mechanical stimuli through specialized mechanosensitive ion channels that convert physical forces into biochemical signals [10].

These cytoskeletal processes establish the biomechanical baseline on which mechanosensitive ion channels act to detect physical forces and modulate motility.

### 3.3. Mechanosensitive Channels and Ionic Signaling

Mechanotransduction in TM is mediated in part by stretch-activated ion channels. Recent findings indicate that Piezo1 not only regulates calcium-dependent relaxation but also participates in redox and cell-death pathways; sustained Piezo1 activation can trigger ferroptosis in TM cells, suggesting that this mechanosensor must operate within a narrow physiological range [10,11]. Piezo1 activation by tensile stretch or shear leads to calcium influx and downstream signaling that modifies arachidonic-acid-derived mediators and relaxes TM contractility, promoting outflow. These findings established Piezo1 as an important molecular link between mechanical stress and TM cellular behavior.

TRPV4 is another mechanically sensitive channel expressed by TM cells; TRPV4 activation regulates calcium-dependent signaling cascades, links to eNOS and NO production in the conventional outflow pathway, and interacts with Rho signaling to influence focal adhesion and cytoskeletal remodeling. Importantly, TRPV4 can be functionally modulated by cytokines: TGF-β_2_ enhances TRPV4-mediated calcium responses and contractility, coupling biochemical and mechanical regulation of TM motility [23]. Impaired TRPV4–eNOS signaling has been shown to alter TM tone and may contribute to dysregulated outflow [20,24]. Recent mechanistic work further shows that mechanosensitive ion channels operate within a broader network of pressure-responsive pathways: pressure/stretch-induced calcium fluxes, local eicosanoid signaling and downstream kinase activation (including ROCK and focal-adhesion kinases) together determine acute and longer-term changes in TM/SC contractility and structural remodeling [25,26]. While mechanotransduction governs acute responses to physical forces, extracellular mediators such as cytokines and growth factors orchestrate longer-term adaptations of the cytoskeleton and ECM composition.

The activity of Piezo1 and TRPV4 therefore sets the ionic and signaling conditions that determine how TM and SC cells respond to extracellular cues such as TGF-β_2_ and ECM composition.

### 3.4. Extracellular Cues, Cytokines and ECM Turnover

Paracrine factors in the aqueous humor profoundly shape TM motility. TGF-β_2_ is a central modulator: elevated levels in glaucomatous eyes promote ECM deposition and alter the balance of matrix metalloproteinases (MMPs) and their inhibitors (TIMPs), shifting the tissue toward matrix accumulation and reduced ECM turnover. Foundational experimental work demonstrated TGF-β_2_-induced ECM production in human TM cells and linked it to outflow resistance [6,27]. At physiological levels, cytokines and growth factors fine-tune matrix remodeling and cell migration; oxidative signals and modest ROS contribute to redox-sensitive modulation of adhesion proteins and actin dynamics, while excessive oxidative stress destabilizes cytoskeletal architecture and impairs motility. The balance between MMP activity and TIMP expression is therefore critical to maintaining a motility-permissive microenvironment in the TM [26,27]. The combined influence of mechanical forces and paracrine signaling converges at the transcriptional level through mechanosensitive pathways such as YAP/TAZ, which integrate cytoskeletal tension into gene-expression programs.

These biochemical influences converge on transcriptional regulators such as YAP/TAZ, which integrate mechanical and cytokine signals into long-term motility programs.

### 3.5. Mechanotransducive Transcriptional Effectors

Mechanical cues are translated not only into acute ionic and cytoskeletal changes but also into longer-term transcriptional programs. A key mediator of this mechanotransduction is the Hippo signaling pathway, a conserved regulator of organ size and tissue homeostasis. In conditions of low mechanical stress, the Hippo pathway is active and phosphorylates its downstream effectors, YAP and TAZ (Transcriptional co-activator with PDZ-binding motif), sequestering them in the cytoplasm for degradation. However, upon sensing ECM stiffness and increased cytoskeletal tension, the Hippo pathway is inactivated. This allows YAP/TAZ to escape phosphorylation, translocate into the nucleus, and partner with transcription factors (like TEADs) to drive the expression of pro-growth and ECM-remodeling genes [28]. This initiates a profibrotic program that, paradoxically, reinforces the very tissue stiffening that activated it. Consequently, the pharmacological modulation of YAP/TAZ activity has emerged as a promising strategy to disrupt this feed-forward loop, restore ECM homeostasis, and promote a motility-permissive phenotype in TM cells [22]. Together, these mechanisms illustrate that cellular motility in the outflow tissues is not dictated by a single pathway but by a dynamic cross-talk among mechanical, biochemical, and transcriptional systems.

This transcriptional output feeds back onto cytoskeletal organization and ECM state, completing the multi-level motility-control loop.

### 3.6. Integrated View: A Dynamic Equilibrium

Taken together, these mechanisms form a continuous motility network in which cytoskeletal tension, mechanosensing, ECM dynamics, and transcriptional feedback operate as a unified system. In summary, healthy cellular movement in the eye’s drainage system is a carefully balanced act. This balance depends on the interplay of several key factors: the tension in the cell’s skeleton and its ability to relax, the rapid signaling of ion channels and the slower process of reshaping the surrounding scaffold, and immediate structural changes versus long-term genetic adaptations. Disruption of any node in this network—overactivation of Rho/ROCK, chronic TGF-β_2_ exposure, impaired mechanosensor function, or ECM stiffening—shifts TM/SC cells toward a less motile, more fibrotic state that reduces outflow and elevates IOP. Recent in vivo imaging confirms that even subtle alterations in TM motion can be detected not only in early stages of primary open-angle glaucoma (POAG) but also in patients with normal-tension glaucoma, underscoring the clinical relevance of physiological motility regulation [29]. Understanding these integrated mechanisms is a prerequisite for mechanistically informed interventions that aim to restore physiological cellular dynamics and outflow function [6,10,18].

**Figure 2:**
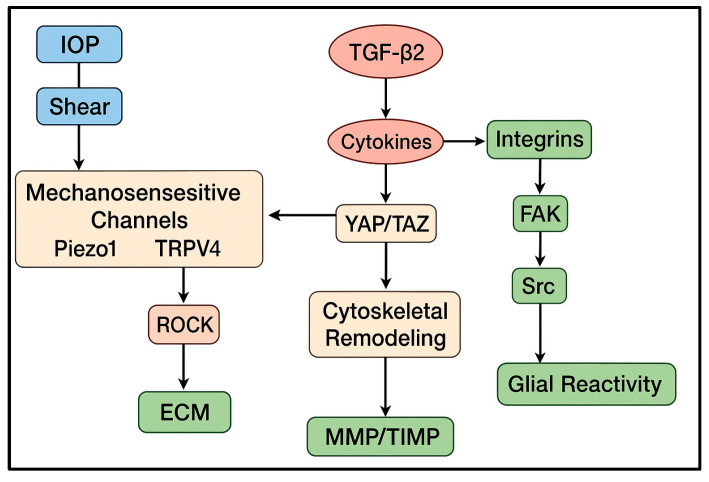
Integrated motility network in glaucoma. Mechanical inputs (IOP, strain, shear) activate mechanosensitive channels (Piezo1, TRPV4), which converge with biochemical regulators (TGF-β_2_, oxidative stress, cytokines) on central cytoskeletal effectors (Rho/ROCK, integrin–FAK, YAP/TAZ). These pathways modulate ECM turnover (MMP/TIMP), adhesion remodeling, contractility, and glial reactivity at the optic nerve head, forming a unified mechanobiological framework for motility failure.

Together, these findings indicate that TM and SC motility is regulated not by isolated pathways but by a highly interconnected motility network. Mechanical cues sensed by Piezo1/TRPV4 converge with biochemical stimuli such as TGF-β_2_, oxidative stress, and cytokines onto shared cytoskeletal nodes including Rho/ROCK signaling, integrin–FAK adhesion dynamics, and the YAP/TAZ transcriptional module. Additional regulators, including NO/eNOS signaling, Wnt components, and microRNA-mediated cytoskeletal control, modulate this core network [21]. This integrated architecture explains why the same pathways (ROCK, TGF-β_2_, mechanosensitive channels) reappear across multiple aspects of glaucomatous remodeling—they form the central hubs through which diverse stimuli influence motility (Figure 2).

## 4. Altered Motility and the Onset of Glaucomatous Remodeling

Glaucoma development begins with a subtle but progressive loss of cellular motility across the aqueous outflow tissues and the optic nerve head (ONH). In primary open-angle glaucoma (POAG), the fine balance between cytoskeletal remodeling, adhesion turnover, and extracellular matrix (ECM) renewal collapses, giving rise to fibrosis, stiffness, and reduced cellular adaptability. These dysfunctions are initiated and sustained by two converging drivers—biochemical stress, particularly TGF-β_2_, and chronic mechanical strain—each reinforcing the other in a self-amplifying loop. The resulting biomechanical and molecular alterations in the trabecular meshwork (TM), Schlemm’s canal (SC), and glial cells of the ONH establish the structural and cellular context for glaucomatous injury.

In the TM and SC, the earliest pathological changes include loss of cellularity, excessive ECM accumulation, and increased tissue stiffness, all of which limit the motile behavior required for aqueous outflow. The stiffened ECM disrupts mechanosensory feedback, locking TM cells in a hypercontractile state and elevating outflow resistance [5,19]. This rigidity reflects both enhanced collagen cross-linking and reduced actin turnover, which together suppress ECM renewal and cytoskeletal plasticity. Among the key biochemical mediators, TGF-β_2_ functions as a master profibrotic switch. Elevated levels in glaucomatous aqueous humor induce synthesis of collagen I/IV, fibronectin, and laminin, and promote cytoskeletal rearrangements such as stress-fiber and cross-linked actin network (CLAN) formation [30]. These structural reinforcements increase cellular rigidity and reduce motility, while downstream activation of RhoA/ROCK and YAP/TAZ pathways links biochemical signaling to mechanical stiffening of the outflow tissue [23,30].

As matrix stiffness rises, mechanical feedback through Piezo1 and TRPV4 channels becomes distorted. Overactivation of these mechanosensors promotes oxidative stress and ferroptotic loss of TM cells, depleting the motile population and amplifying fibrotic remodeling [11,23]. Endothelial-to-mesenchymal transition (EndMT) of SC endothelial cells further contributes to rigidity: under chronic TGF-β_2_ exposure, SC cells lose junctional integrity and vacuolar organization while acquiring α-SMA- and fibronectin-rich mesenchymal features [14]. The resulting decline in permeability completes the mechanical bottleneck that limits outflow. Importantly, ROCK inhibition can prevent this transition and partially restore motility, suggesting pharmacologic reversibility of these changes [31]

Recent advances in high-resolution imaging confirm that these mechanical and motility alterations appear early. Phase-sensitive optical coherence tomography (OCT) reveals that TM motion amplitude is already reduced in early or normal-tension glaucoma [29], while volumetric OCT elastography now enables real-time mapping of TM deformation and stiffness in vivo [32]. These findings indicate that motility impairment precedes overt pressure elevation, positioning it as a primary pathogenic event rather than a secondary response.

### Glial and Optic Nerve Head Remodeling

Parallel to anterior-segment remodeling, glial motility in the ONH undergoes maladaptive transformation. Astrocytes exposed to chronic pressure or biochemical stress shift from a quiescent, supportive phenotype to a reactive, contractile state characterized by migration, ECM deposition, and altered adhesion [33]. This reactive astrocytosis is now recognized not merely as a secondary event but as an initiating component of optic nerve pathology [34]. Within the lamina cribrosa (LC), reactive astrocytes overexpress GFAP, vimentin, and integrin-FAK signaling molecules while secreting collagen IV, laminin, and tenascin-C. The resulting ECM expansion stiffens the ONH and restricts axonal transport. Astrocyte-derived TGF-β_2_ and Connective Tissue Growth Factor (CTGF) reinforce these profibrotic processes, generating a local microenvironment of rigidity and metabolic compromise.

Single-cell analyses reveal that astrocytic reactivity is heterogeneous and graded: some subpopulations transiently adopt motile or phagocytic roles, whereas others drive fibrosis and inflammation [13]. Early-reactive astrocytes may protect retinal ganglion cell (RGC) axons, but sustained activation converts them into scar-forming cells that inhibit regeneration. This dynamic imbalance between adaptive and maladaptive glial motility bridges mechanical and neurodegenerative paradigms of glaucoma, establishing the ONH as both target and amplifier of disease progression.

**Figure 3:**
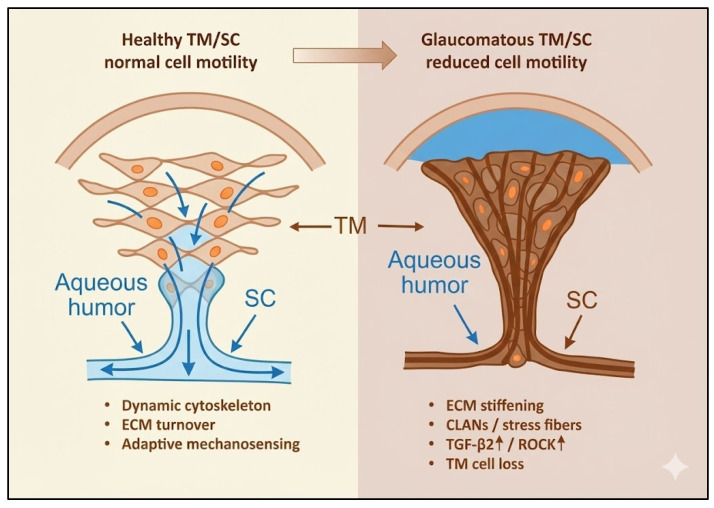
Healthy versus Sclerotic Trabecular Meshwork and Schlemm’s Canal. Left: Healthy trabecular meshwork (TM) and Schlemm’s canal (SC). The normal TM displays a loose, interconnected lamellar architecture composed of compliant beams and motile TM cells. This structure maintains low outflow resistance by permitting dynamic deformation and facilitating the passage of aqueous humor (AH) toward the SC. The SC lumen remains widely patent, ensuring efficient drainage of AH and stable intraocular pressure. Right: Diseased, sclerotic trabecular meshwork in glaucoma. Chronic mechanical and biochemical stress (TGF-β_2_ elevation, ROCK hyperactivation, ECM stiffening) leads to TM sclerosis, loss of cellularity, cross-linked actin network (CLAN), and deposition of dense extracellular matrix. The tissue becomes rigid and compacted, collapsing the juxtacanalicular space and narrowing the SC lumen. These structural alterations markedly impair AH outflow, contributing to increased outflow resistance and ocular hypertension.

Together, alterations in TM/SC and ONH glial motility delineate a continuous pathogenic axis—from impaired outflow to axonal degeneration—linking mechanical stress, fibrosis, and neuroglial dysfunction within a single mechanobiological continuum (Figure 3). Although ONH remodeling is driven more by reactive glial plasticity than by classical cell migration, these cytoskeletal and adhesion-dependent changes still represent a form of altered motility relevant to glaucomatous damage. Astrocytes subjected to chronic stress reorient their processes, modify integrin–FAK signaling, and deposit ECM components that stiffen the lamina cribrosa. These changes do not require the same degree of motile behavior seen in TM cells but are functionally analogous in that they remodel tissue structure and alter biomechanics.

## 5. Interplay of Mechanical Forces and Motility

Mechanical stress is both a regulator and a consequence of motility loss. In the healthy eye, cyclic stretch, shear, and substrate deformation due to IOP variations continuously tune cytoskeletal tension and adhesion turnover within TM and SC cells. When these mechanical cues become excessive or chronic, they transform adaptive responses into maladaptive rigidity, coupling biomechanics directly to glaucoma pathogenesis.

TM and SC cells act as mechanoresponsive units that translate physical deformation into calcium-dependent signaling. Among the key mediators, Piezo1 and TRPV4 channels orchestrate the response to stretch and shear, regulating cytoskeletal remodeling and aqueous outflow [35]. Under normal conditions, transient channel activation promotes cytoskeletal flexibility and balanced contractility; however, chronic mechanical loading or fibrotic stiffening leads to sustained activation, generating calcium overload, oxidative stress, and ferroptotic death of TM cells [11]. TRPV4 overactivation, especially in TGF-β_2_-enriched environments, further enhances cellular contractility and elevates IOP [23]. Mechanosensitive channels thus act as dual regulators—facilitating adaptive motility under physiological strain but driving degeneration when persistently overstimulated.

Matrix stiffness is not merely a structural outcome but a key signal that governs cellular phenotype. In glaucoma, accumulation of cross-linked collagen and fibronectin alters focal-adhesion maturation, maintaining RhoA/ROCK and YAP/TAZ activation. This feedback loop traps TM and SC cells in a perpetually contractile state, further reducing motility. Direct mechanical probing and imaging analyses confirm that glaucomatous TM is several-fold stiffer than healthy tissue [5,19], and reduced TM compliance detected by motion-tracking OCT indicates that mechanical stiffening precedes clinical pressure rise [29]. These alterations in TM/SC motility arise from coordinated dysregulation of the motility network rather than from single pathways acting independently.

The convergence of mechanical strain, mechanosensor activity, and cytoskeletal feedback defines a single, self-regulating system that maintains outflow homeostasis. When this system becomes chronically biased toward stiffness, motility collapse follows. Recognition of this mechanobiological failure has inspired therapies aimed at restoring cytoskeletal flexibility—most notably Rho-kinase inhibitors (ROCKi)—which relax actomyosin tension and enhance TM/SC compliance [18,36]. This mechanistic understanding bridges basic biomechanics with therapeutic modulation, forming the conceptual foundation for regenerative and pharmacologic strategies discussed in later chapters.

## 6. Consequences of Impaired Motility

Thus, impaired TM motility establishes the biomechanical conditions that increase ONH susceptibility, linking anterior-segment stiffness with posterior-segment glial remodeling. Loss of cellular motility disrupts the homeostatic equilibrium of the eye, triggering a cascade of biomechanical, metabolic, and neurodegenerative changes. In glaucoma, defective remodeling in the TM and SC elevates IOP, while maladaptive glial motility in the ONH accelerates axonal loss and limits repair.

When TM and SC cells lose cytoskeletal flexibility, the outflow pathway stiffens, impairing its ability to adjust to pressure fluctuations. ECM accumulation, actin reinforcement, and cell depletion produce a rigid, non-responsive network that elevates IOP [19,30]. High-resolution imaging confirms reduced TM motion amplitude even before pressure rise [29]. At the molecular level, chronic TGF-β_2_ exposure and Piezo1/TRPV4 overactivation maintain this rigidity and induce ferroptotic cell loss [11,23]. The resulting feed-forward cycle—where increased stiffness raises pressure and pressure accelerates stiffness—constitutes the mechanical engine of glaucoma.

Elevated mechanical load and glial remodeling transform the ONH into a progressively fibrotic structure. Astrocytes, driven by mechanical strain and local cytokines, reorganize their cytoskeleton and ECM output, compromising axonal transport and trophic support [33]. Single-cell studies reveal temporally distinct astrocyte populations: early-reactive, potentially protective cells, and late, fibrotic scar-forming ones [13,37]. Failure of the lipoxin B_4_ pathway enhances this chronic activation and reduces neurotrophic signaling [38]. These events link mechanical stress to intrinsic neuronal vulnerability, suggesting that glaucoma progression reflects both biomechanical and neuroglial maladaptation [34].

### Impaired Repair and Regeneration

As motility declines, the capacity for structural and functional recovery is lost. TM progenitor cells, normally able to migrate and repopulate damaged regions, become trapped by fibrotic ECM [15]. Similarly, ONH scarring and chronic inflammation create inhibitory ECM barriers that block axonal regrowth. Glial contractility that once supported repair becomes a fixed fibrotic response, locking the tissue into a degenerative state. Collectively, these mechanisms define glaucoma as a disease of motility failure and biomechanical entrapment, where pressure elevation and neural loss are downstream manifestations of a deeper collapse in cellular adaptability.

**Figure 4:**
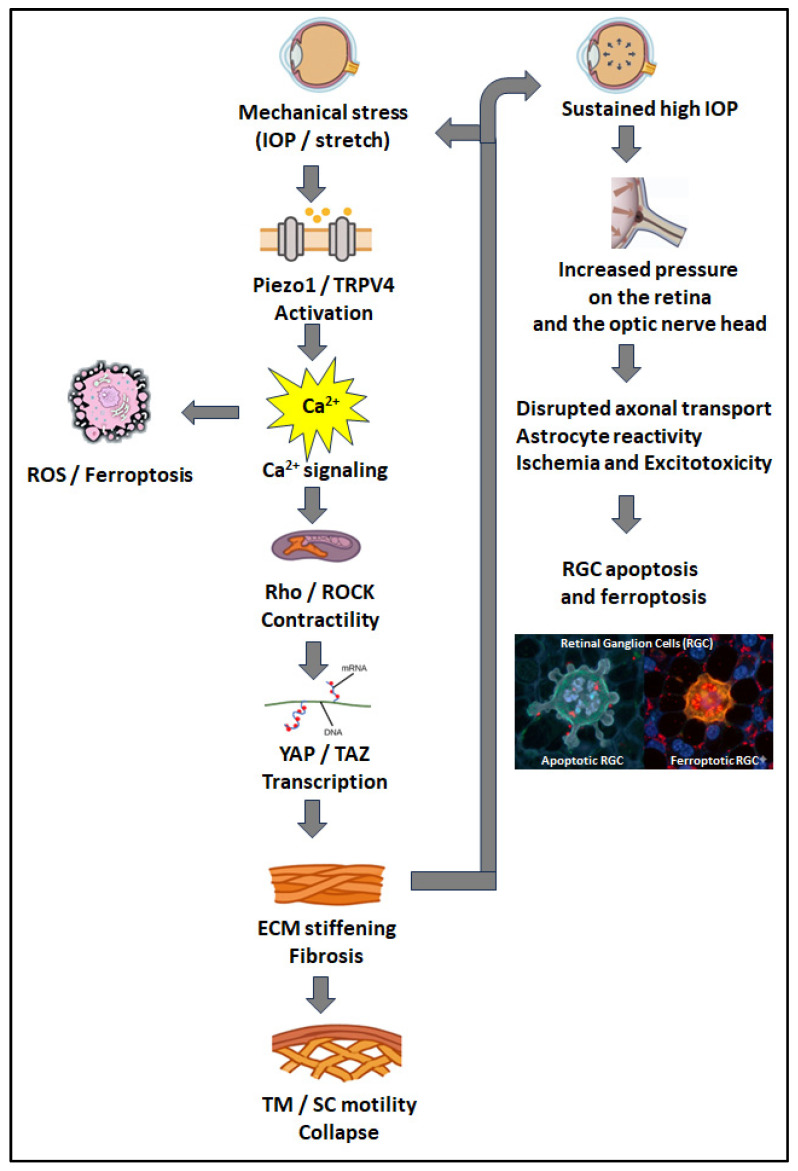
Mechanotransduction and Dysregulated Motility in Glaucoma Pathogenesis. This schematic illustrates how mechanical stress drives glaucomatous damage through aberrant cellular motility. In the anterior segment, IOP-mediated activation of Piezo1/TRPV4 channels initiates a Ca^2+^ and Rho/ROCK signaling cascade, promoting pathological actomyosin contractility and YAP/TAZ-driven transcription. This leads to ECM stiffening and, critically, a loss of normal TM and SC cell motility, causing aqueous outflow pathway collapse and sustained high IOP. This elevated pressure injures the posterior segment, where impaired axonal transport in RGCs and reactive astrocyte changes further highlight motility deficits. These insults, combined with ischemia and excitotoxicity, ultimately trigger RGC death via apoptosis and ferroptosis, resulting in irreversible vision loss.

Summarizing, glaucoma evolves through a sequential disruption of motility-dependent homeostasis that unites the anterior and posterior segments into a single pathogenic continuum. Initial biochemical stress (notably TGF-β_2_) and mechanical strain impair TM and SC cell motility, leading to ECM accumulation, tissue stiffening, and reduced outflow adaptability. This rigidity elevates intraocular pressure, reinforcing fibrosis and amplifying mechanosensitive dysregulation through Piezo1 and TRPV4. Persistent stiffness and cell loss extend their impact posteriorly, where reactive astrocyte motility in the ONH drives lamina cribrosa fibrosis, axonal compression, and neuroglial imbalance. As these processes progress, repair mechanisms fail, transforming transient stress responses into irreversible remodeling and degeneration. This stepwise cascade—from impaired motility to fibrosis, pressure elevation, and neurodegeneration—defines the mechanobiological trajectory of glaucoma and provides the conceptual foundation for the therapeutic strategies explored in the following chapter (Figure 4).

## 7. Therapeutic Restoration of Motility and Future Directions

Glaucomatous pathology is fundamentally driven by a failure of motile and cytoskeletal responses, which impairs trabecular meshwork (TM) compliance and aqueous outflow, elevating intraocular pressure (IOP) [39,40]. This dysfunction may also contribute to a loss of optic nerve head resilience independent of IOP, a key feature of normal-tension glaucoma [41]. Consequently, therapeutic strategies are increasingly focused on restoring cellular motility, remodeling capacity, and mechanical adaptability by targeting the molecular systems that integrate cytoskeletal tension, extracellular matrix (ECM) composition, and mechanotransduction.

### 7.1. Pharmacologic Modulation: Rho/ROCK Inhibition

The Rho/ROCK axis is a central regulator of actomyosin contractility. In glaucoma, its chronic activation sustains a hypercontractile, low-motility state that increases outflow resistance [18,42]. ROCK inhibitors (ROCKi) directly counter this by relaxing actomyosin tension, disassembling stress fibers, and restoring cellular motility and permeability in TM and SC cells [14]. This mechanistic rationale has been clinically validated. The MERCURY Phase 3 trials and the broader ROCKET program provided foundational evidence for netarsudil’s sustained IOP-lowering efficacy [36], while the J-ROCKET trial confirmed this as a class effect by demonstrating non-inferiority between netarsudil and ripasudil [43]. Beyond IOP reduction, ROCK inhibition exerts anti-fibrotic effects, such as suppressing TGF-β_2_-induced EndMT in SC cells, thereby preserving a functional, motile endothelial phenotype [14]. The main challenge for this drug class is off-target effects like conjunctival hyperemia, driving research into isoform-selective ROCK inhibitors and tissue-targeted delivery systems to improve specificity [36,44].

### 7.2. Regenerative and Cell-Based Strategies

A complementary approach aims to repopulate the TM itself. The existence of resident stem/progenitor cells suggests an intrinsic capacity for self-repair, which is overwhelmed in glaucoma [45]. Transplantation of iPSC-derived or TM-like cells can restore aqueous outflow in models by integrating into the native meshwork and remodeling the pathologic ECM [46,47,48].

A key challenge for these therapies is ensuring transplanted cells can migrate and integrate within the stiff, fibrotic TM environment. Innovative solutions are emerging, such as magnetically guided cell delivery to enhance homing and retention [49]. Furthermore, combining regenerative approaches with ROCK inhibition may create a more motility-permissive niche by preconditioning the matrix and enhancing engraftment [31,49].

### 7.3. Emerging Targets and Multi-Modal Strategies

Other targets offer additional avenues to restore motility. Direct modulation of the TGF-β_2_ pathway—via mTOR inhibitors or by targeting its downstream microRNA network—can mitigate fibrogenic responses and abnormal cytoskeletal organization [50,51]. Similarly, mechanosensitive ion channels like Piezo1 and TRPV4 represent promising targets, as their overactivation under strain can lead to calcium overload and ferroptotic cell death, depleting the motile TM cell pool [11].

The long-term durability of any intervention is challenged by a persistent “fibrotic memory” and continuous mechanical stress. Sustaining biomechanical homeostasis may therefore require intermittent or combination therapies that integrate cytoskeletal relaxation (ROCKi), anti-fibrotic modulation, and ferroptosis prevention [1,11,52].

### 7.4. Diagnostic Innovation and Future Paradigms

A critical frontier is the early detection of motility dysfunction, which may precede overt IOP elevation. Emerging imaging technologies are making this possible. Optical coherence elastography (OCE) and ultra-high-speed volumetric OCT now enable direct, in vivo assessment of TM stiffness and dynamic motion [32,53]. Clinical studies have confirmed that glaucomatous eyes exhibit increased ocular rigidity and reduced TM motion [29,54]. The integration of these modalities with AI analytics could enable a predictive, motility-centered diagnostic paradigm, shifting management from a purely pressure-based model to one that directly monitors and targets tissue biomechanics.

### 7.5. Concluding Perspective

The evidence consolidated here confirms that targeting the cellular motility deficit offers a powerful, direct strategy to counteract glaucomatous outflow pathology. From pharmacologic relaxation of the cytoskeleton to the regenerative restoration of the cellular architecture, these approaches collectively aim to re-establish the mechanical adaptability of the conventional outflow pathway. The success of this new therapeutic axis will depend on the parallel development of advanced diagnostics to guide patient-specific application.

## 8. Conclusions

The investigation of cellular motility and mechanobiology culminates in a fundamental redefinition of glaucoma. It is not solely a disease of pressure, but a disorder of impaired adaptability, where the critical capacities for cytoskeletal dynamism, ECM remodeling, and cellular resilience fail across the anterior and posterior segments. This unified perspective seamlessly links the pathological stiffening of the TM to the compromised structural support of the ONH, revealing a continuous biomechanical pathway to RGC vulnerability. While motility dysregulation is mechanistically most prominent in the TM, ONH glial remodeling represents a downstream extension of the same biomechanical stress pathways and as such, it has been incorporated into the present review.

Within this framework, IOP elevation is seen as a primary symptom of upstream mechanical failure at the TM, rather than an isolated cause. This paradigm shift is actively reshaping the therapeutic landscape, moving beyond palliation towards functional restoration. The strategies detailed in this review—from ROCK inhibition and regenerative cell therapy to the targeting of specific mechanosensory and fibrotic pathways—all converge on a common objective: to reactivate the tissue’s innate ability to sense, respond to, and withstand mechanical stress. The ultimate clinical goal is now twofold: to lower IOP and, more importantly, to re-establish the cellular motility and tissue resilience that constitute physiological ocular homeostasis.

Looking forward, the promise of this new paradigm hinges on integration. The convergence of novel biologic therapies with non-invasive imaging technologies capable of quantifying tissue stiffness, cellular motility, and metabolic stress in vivo will be transformative. This will enable a shift from a reactive, pressure-centric model to a predictive, mechanism-based practice. In the future, interventions will be deployed preemptively, guided by an individual’s specific biomechanical and cellular profile, to preserve the adaptable nature of the eye and prevent the irreversible loss of visual function. Restoring motion might lead to restoring sight.

## Data Availability

No new data were created or analyzed in this study. Data sharing is not applicable to this article.
